# Research on Navigation Method for Subsea Drilling Robot Based on Inertial Navigation and Odometry

**DOI:** 10.3390/s26082457

**Published:** 2026-04-16

**Authors:** Yingjie Liu, Peng Zhou, Feng Xiao, Chenyang Li, Junhui Li, Jiawang Chen, Ziqiang Ren

**Affiliations:** 1 Hangzhou Universal Control Mechatronical Engineering LLC, Hangzhou 310005, China; yingjieliu_wl@163.com; 2 Donghai Laboratory, Zhejiang University, Zhoushan 316021, China; 3 Ocean College, Zhejiang University, Zhoushan 316021, China; 12334035@zju.edu.cn (P.Z.);; 4 Hainan Institute, Zhejiang University, Sanya 572000, China

**Keywords:** subsea drilling robot, robust square-root cubature Kalman filter, slippage compensation, non-Gaussian noise, integrated navigation

## Abstract

This paper proposes a robust navigation method based on a robust square-root cubature Kalman filter (RSRCKF) to address the accuracy divergence of integrated navigation systems caused by drilling-induced slippage and the mismatch between the tail-cable encoder and the robot motion during operations of a seafloor drilling robot in deep-sea soft sedimentary layers. Considering the large-deformation mechanical characteristics of the seabed under drilling conditions, a unified state-space model incorporating a time-varying odometer scale-factor error is first established. To alleviate the numerical instability of the nonlinear system in the presence of non-Gaussian noise, a square-root cubature Kalman filter (SRCKF) framework is employed, in which the positive definiteness of the error covariance matrix is dynamically preserved via QR decomposition. Subsequently, an online fault detection mechanism based on a modified chi-square test is developed. By introducing a two-segment IGG (a classical robust weighting scheme) weighting function, an adaptive variance inflation factor is constructed to enable real-time identification and down-weighting of abnormal observations induced by slippage. Field experiments, including drilling and turning tests conducted on tidal mudflats off the coast of Zhoushan, demonstrate that the proposed method can effectively mitigate the impact of “false displacement” disturbances caused by typical soft clay slippage conditions through enhanced statistical robustness. Taking the conventional SINS/OD integration scheme as the baseline, the proposed method achieves an approximate 82.4% reduction in positioning error. These results verify the robustness and engineering applicability of the proposed algorithm in complex seabed environments.

## 1. Introduction

With the increasing demand for marine energy exploration, particularly for deep-sea resources such as natural gas hydrates [1,2,3], seabed drilling robots have become indispensable for in situ sampling and detection in sedimentary environments. Compared with conventional underwater vehicles, drilling robots operate in direct contact with deformable seabed media, where complex interaction forces and terrain variability significantly affect their motion characteristics. Moreover, these systems must function under extreme conditions, including high pressure, the absence of GNSS signals, and severely limited underwater communication, which pose substantial challenges to achieving reliable and continuous navigation. In underwater navigation, strapdown inertial navigation systems (SINS) integrated with Doppler velocity logs (DVLs) are widely adopted due to their complementary characteristics. However, during drilling operations, sediment disturbance and turbidity often lead to acoustic degradation or even beam lock loss in DVL measurements [4,5], making such configurations unreliable in practical scenarios. As an alternative, odometers or tail-cable encoders are commonly employed to provide auxiliary motion information for SINS [6]. Nevertheless, unlike rigid-ground environments, soft seabed sediments such as clay and silt introduce significant nonlinear slippage, resulting in discrepancies between encoder measurements and actual motion. This slippage effect leads to observation errors that exhibit strong non-Gaussian and heavy-tailed characteristics. As a result, conventional Kalman filter (KF)- and extended Kalman filter (EKF)-based methods, which rely on Gaussian noise assumptions, may suffer from severe performance degradation or even divergence under such conditions [7,8,9]. To address nonlinear system dynamics, advanced nonlinear filtering approaches such as the cubature Kalman filter (CKF) have been proposed [10], and further improvements have been achieved through robust estimation techniques and adaptive noise modeling [11,12,13]. While these methods enhance robustness against outliers to some extent, they remain insufficient for seabed drilling applications. Specifically, existing approaches exhibit three main limitations. First, they often lack the capability to simultaneously handle abrupt disturbances (e.g., transient slippage) and slowly varying systematic errors. Second, many robust filtering methods rely on fixed or heuristic weighting strategies, which cannot adapt effectively to time-varying environmental conditions. Third, conventional constraint-based methods typically employ rigid kinematic constraints, such as non-holonomic constraints (NHCs), without considering the variability of constraint validity in deformable terrains, thereby limiting their practical applicability [14,15,16,17,18,19].

To address the above challenges, this paper proposes a robust and adaptive navigation framework tailored for seabed drilling robots operating in soft sediment environments. A unified error state model with time-varying scale factors is first developed to better capture the stochastic characteristics of slippage-induced errors. On this basis, a robust square-root cubature Kalman filter (SRCKF) is designed by integrating adaptive statistical testing and soft constraint mechanisms. These strategies enable real-time suppression of outliers and dynamic adjustment of constraint weights, thereby improving both robustness and adaptability under complex and time-varying conditions. The main contributions of this work are summarized as follows:

1. An adaptive variance inflation mechanism is constructed to enable real-time detection and down-weighting of slippage-induced abnormal observations.
2. A robust SRCKF algorithm with adaptive statistical testing is developed to effectively mitigate non-Gaussian disturbances and prevent filter divergence.
3. A soft constraint mechanism is introduced to dynamically adjust the influence of NHC according to terrain conditions, enhancing the flexibility and reliability of constraint utilization.

## 2. Structure and Modeling of the Submarine Drilling Robot

In order to meet the requirements of structural compactness and operational robustness for drilling robots in confined subsea environments, a compact mechanical configuration is developed. Based on this configuration, the kinematic and dynamic characteristics of the system are modeled, which provide a theoretical foundation for subsequent navigation solutions. Furthermore, non-holonomic constraints (NHCs) and zero-velocity updates (ZUPTs) are incorporated to enhance the accuracy and stability of dead reckoning during drilling operations.

### 2.1. Structure of Miniaturized Underwater Profiler

The seabed stratum drilling robot is designed to operate in high-pressure, low-visibility, and highly disturbed environments [20], where long-duration autonomous navigation must be achieved without GNSS and with minimal external references. The system adopts a two-tier architecture comprising a seabed base station and a drilling robot. The base station provides deployment, retrieval, power, and communication support, while the robot integrates control, drive, data acquisition, and sensing modules to enable autonomous subsea drilling operations.

**Figure 1:**
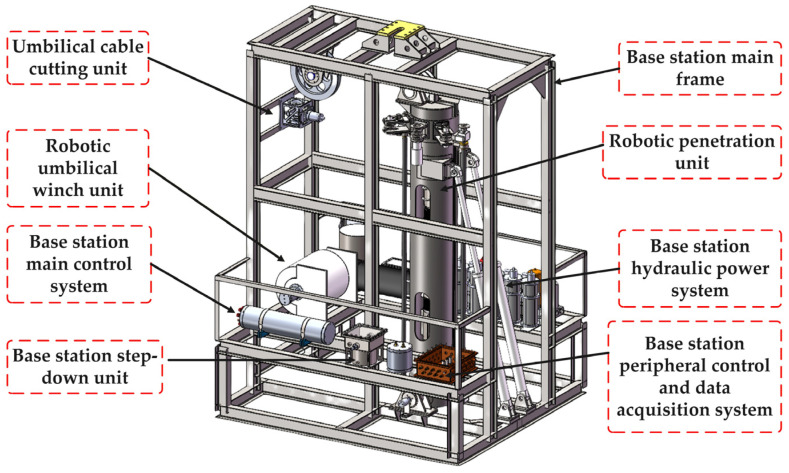
Overall structure model of submarine base station.

Figure 1 shows the overall structure of the submarine base station. The system is built on a main frame and integrates multiple functional modules. The robotic penetration unit is centrally mounted, while the hydraulic power system provides actuation support. The main control system and peripheral control and data acquisition system coordinate operation and data processing.

**Figure 2:**
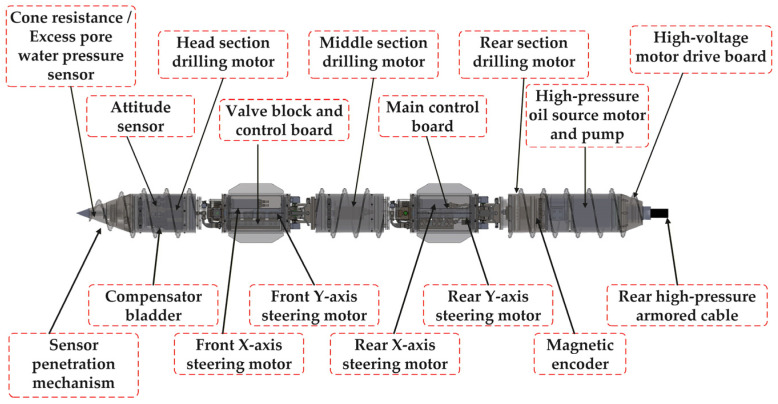
Overall structure model of submarine bottom drilling robot.

Figure 2 shows the overall structure of the submarine bottom drilling robot. The system adopts a modular configuration along the longitudinal axis. The front section integrates a cone resistance/excess pore water pressure sensor and a penetration mechanism for in situ sediment measurement, along with an attitude sensor for orientation estimation. The drilling unit consists of head, middle, and rear motors, providing sufficient penetration capability. Steering motors along the *x*-axes and *y*-axes at both the front and rear enable trajectory control. The central module includes the main control board and valve block, coordinating system operation, while a high-pressure motor–pump unit supplies hydraulic power. A compensator bladder is used for pressure balance.

### 2.2. Error Model of Inertial/Odometry Combined Navigation

High-precision integrated navigation fundamentally depends on accurately characterizing subsystem error propagation and their coupled dynamics under operational conditions. For seabed drilling robots operating in deep-sea soft sediments, raw sensor measurements alone are inadequate for long-term positioning. This section integrates robust filtering theory with seabed mechanical characteristics to establish a unified state-space model that captures SINS and odometer (OD) errors under soft mud-induced slippage. In addition, the physical validity limits of non-holonomic constraints (NHCs) and zero-velocity updates (ZUPTs) during drilling operations are systematically analyzed.

#### 2.2.1. Coordinate System Definition

The inertial coordinate system is abbreviated as i-frame ($Ox_{i}y_{i}z_{i}$). The origin of the coordinate system is located at the Earth’s center. This coordinate system is absolutely stationary and does not rotate with the Earth. The $x_{i}$ and $y_{i}$ axes lie in the Earth’s equatorial plane, with the $x_{i}$ axis directed toward the vernal equinox, while the $y_{i}$ axis aligns with the Earth’s rotation axis and points toward the North Pole.

The Earth coordinate system is abbreviated as e-system ($Ox_{e}y_{e}z_{e}$). The origin of the coordinate system is located at the Earth’s center. This coordinate system rotates with the Earth. The $x_{e}$ and $y_{e}$ axes lie in the Earth’s equatorial plane, with the $x_{e}$ axis pointing toward the intersection of the equator and the prime meridian (Greenwich), and the $z_{e}$ axis aligned with the Earth’s rotation axis, pointing toward the North Pole.

The inertial measurement coordinate system is abbreviated as b-system ($Ox_{b}y_{b}z_{b}$). The origin of the coordinate system is located at the center of gravity of the carrier. The coordinate axis direction is related to the motion direction of the carrier. The $x_{b}$, $y_{b}$, and $z_{b}$ axes are aligned with the Drilling robot’s right, forward, and upward directions.

The odometry carrier coordinate system is abbreviated as m-system ($Ox_{m}y_{m}z_{m}$). The origin of the coordinate system is defined at the connection point between the odometer traction gear and the track, according to the practical configuration of the drilling robot. The $x_{m}$ is perpendicular to the forward direction of the drilling robot, the $y_{m}$ points along the forward direction of the drilling robot, and the $z_{m}$ is perpendicular to the carrier plane.

The navigation coordinate system is abbreviated as n-system ($Ox_{n}y_{n}z_{n}$). The origin of the coordinate system is located at the center of gravity of the drilling robot. The geographical location of the drilling robot (longitude φ, latitude λ) determines the orientation of this coordinate system. The $x_{n}$, $y_{n}$, and $z_{n}$ axes point toward the east, north, and sky directions.

Scalars are denoted by italic letters, vectors by bold letters, and matrices by bold uppercase letters. The superscripts ${\left(\cdot \right)}^{n}$ and ${\left(\cdot \right)}^{b}$ denote quantities expressed in the navigation frame and body frame, respectively. The operator δ(·) represents the error state of a variable; the notation diag(·) denotes a diagonal matrix.

#### 2.2.2. Error Model of Inertial Navigation System

Inertial navigation systems (INS) are classified as inferential navigation, which infers the position of the next point from the position of a known point based on continuous measurements of the heading angle and velocity of a moving object. Therefore, the current position of a moving object can be continuously determined. A coordinate system is established based on the measurement axis of the gyroscope in the INS, and the gyroscope provides the direction and attitude angle of the moving object. The accelerometer and the gyroscope share the same measurement axis. The acceleration of the moving object is measured by the accelerometer. The velocity is obtained by integrating the velocity over time once, and the displacement is obtained by integrating the velocity over time once [21,22,23,24].

The attitude error vector in the navigation frame is defined as

(1) $$\varphi ={\left[\begin{array}{ccc} \delta \varphi_{E} & \delta \varphi_{N} & \delta \varphi_{U} \end{array}\right]}^{T}$$

where $\varphi_{E}$, $\varphi_{N}$ and $\varphi_{U}$ denote the small-angle attitude error vectors, which are referred to as east, north, and sky attitude angle errors, respectively. Based on the SINS error propagation mechanism, the attitude error dynamics can be formulated as

(2) $$\dot{\varphi }=-\omega_{in}^{n}\times \varphi +\delta \omega_{in}^{n}-c_{b}^{n}\varepsilon^{b}$$

where $\omega_{in}^{n}$ denotes the angular velocity of the navigation frame with respect to the inertial frame, expressed in the navigation frame, which is given by

(3) $$\omega_{in}^{n}=\omega_{ie}^{n}+\omega_{en}^{n}$$

The Earth rotation rate expressed in the navigation frame is defined as

(4) $$\omega_{ie}^{n}={\left[0\;\omega_{ie}\cos\left(\lambda \right)\;w_{ie}\sin\left(\lambda \right)\right]}^{T}$$

The transport rate is given by

(5) $$\omega_{en}^{n}={\left[\begin{array}{ccc} -\frac{v_{n}}{R_{n}+h} & \frac{v_{e}}{R_{e}+h} & \frac{v_{e}\tan\left(\lambda \right)}{R_{e}+h} \end{array}\right]}^{T}$$

where λ denotes the latitude, $v_{n}$ and $v_{e}$ represent the vehicle velocities in the north and east directions, respectively, $R_{n}$ and $R_{e}$ denote the meridian and transverse radii of curvature, respectively, and h is the altitude.

The gyroscope bias in the body frame is defined as

(6) $$\varepsilon^{b}={\left[\varepsilon_{x}\;\varepsilon_{y}\;\varepsilon_{z}\right]}^{T}$$

where $\varepsilon_{x}$, $\varepsilon_{y}$, and $\varepsilon_{z}$ denote the bias components along the three axes.

The transformation matrix from the body frame to the navigation frame is denoted by $C_{b}^{n}$:

(7) $$C_{b}^{n}=\left[\begin{array}{ccc} \cos\left(\psi \right)\cos\left(\gamma \right)+\sin\left(\psi \right)\sin\left(\gamma \right)\sin\left(\theta \right) & \sin\left(\gamma \right)\cos\left(\theta \right) & \sin\left(\psi \right)\cos\left(\gamma \right)-\cos\left(\psi \right)\sin\left(\gamma \right)\sin\left(\theta \right)\\ -\cos\left(\psi \right)\sin\left(\gamma \right)+\sin\left(\psi \right)\cos\left(\gamma \right)\sin\left(\theta \right) & \cos\left(\gamma \right)\cos\left(\theta \right) & -\sin\left(\psi \right)\sin\left(\gamma \right)-\cos\left(\psi \right)\cos\left(\gamma \right)\sin\left(\theta \right)\\ -\sin\left(\psi \right)\cos\left(\theta \right) & \sin\left(\theta \right) & \cos\left(\psi \right)\cos\left(\theta \right) \end{array}\right]$$

where ψ represents the heading angle, γ represents the roll angle, and θ represents the pitch angle.

According to the specific force equation of inertial navigation, the velocity error dynamics can be expressed as

(8) $$\delta {\dot{v}}^{n}=f^{n}\times \varphi -\left(2\omega_{ie}^{n}+\omega_{en}^{n}\right)\times \delta v^{n}+v^{n}\times \left(2\delta \omega_{ie}^{n}+\delta \omega_{en}^{n}\right)+c_{b}^{n}\sigma^{b}$$

where $\delta v^{n}={\left[\delta v_{e}^{n}\;\delta v_{n}^{n}\;\delta v_{u}^{n}\right]}^{T}$ is the velocity error vector and $f^{n}={\left[f_{e}\;f_{n}\;f_{u}\right]}^{T}$ denotes the specific force expressed in the navigation frame. $v^{n}={\left[v_{e}\;v_{n}\;v_{u}\right]}^{T}$ represents the velocity vector in the navigation frame. The accelerometer bias in the body frame is denoted as $\sigma^{b}={\left[\sigma_{x}\;\sigma_{y}\;\sigma_{z}\right]}^{T}.$ The term ($C_{b}^{n}{\;f}^{b}$) introduces coupling between attitude errors and velocity errors.

The position error equation is expressed by Taylor expansion as

(9) $$\left[\begin{array}{c} \delta \dot{L}\\ \delta \dot{\lambda }\\ \delta \dot{h} \end{array}\right]=\left[\begin{array}{ccc} 0 & \frac{1}{R_{n}+h} & 0\\ \frac{\sec L}{R_{n}+h} & 0 & 0\\ 0 & 0 & 1 \end{array}\right]\left[\begin{array}{c} \delta v_{e}\\ \delta v_{n}\\ \delta v_{u} \end{array}\right]+\left[\begin{array}{ccc} 0 & 0 & -\frac{v_{N}}{{\left(R_{e}+h\right)}^{2}}\\ \frac{v_{e}\sec\mathrm{Ltan}L}{R_{n}+h} & 0 & -\frac{v_{e}\sec L}{{\left(R_{n}+h\right)}^{2}}\\ 0 & 0 & 0 \end{array}\right]\left[\begin{array}{c} \delta L\\ \delta \lambda \\ \delta h \end{array}\right]$$

#### 2.2.3. Odometer Error Model

The odometer estimates the carrier’s forward velocity by measuring wheel rotations over a fixed sampling interval. The resulting velocity in the body frame is denoted as $v_{odo}^{b}$. Owing to the kinematic configuration of the system—where the tail composite cable is constrained by the base station pulley and maintains tension via spring preload—the encoder effectively measures motion along a single axis. Consequently, the lateral and vertical velocity components are negligible, and the odometer output in the body frame satisfies a uniaxial velocity assumption.

Under ideal conditions, the velocity of the drilling robot in the body coordinate system can therefore be expressed as

(10) $$v_{odo}^{b}=[0\;{\;v}_{odo-y}^{b}\;0{]}^{T}$$

In practical applications, the odometer measurements are affected by scale factor errors and installation misalignment. Let $\delta_{K}$ denote the scale factor error, and let α denote the installation misalignment vector, defined as

(11) $$\alpha ={\left[\begin{array}{ccc} \alpha_{\theta } & \alpha_{\gamma } & \alpha_{\psi } \end{array}\right]}^{T}$$

The measured forward velocity can be expressed as

(12) $$v_{odo}^{n}=\left(\begin{array}{c} I-\varphi \times \end{array}\right)C_{b}^{n}\left(\begin{array}{c} I-\alpha \times \end{array}\right)\left(\begin{array}{c} 1+\delta K \end{array}\right)v_{odo}^{b}$$

Linearizing (12) yields the odometer error model:

(13) $$\delta v_{odo}^{n}=v_{odo}^{n}\times \varphi +v_{odo}^{m}\left[\begin{array}{ccc} -C_{13} & C_{12} & C_{11}\\ -C_{23} & C_{22} & C_{21}\\ -C_{33} & C_{23} & C_{31} \end{array}\right]{\left[\begin{array}{ccc} \alpha_{\theta } & \delta K & \alpha_{\psi } \end{array}\right]}^{T}$$

$v_{odo}^{m}$ denotes the velocity at the odometer installation point. Considering that the odometer and the inertial navigation unit are typically mounted at different locations, $v_{odo}^{b}$ represents the odometer velocity transformed into the body coordinate frame, whose origin is located at the inertial navigation unit.

The conversion relationship between $v_{odo}^{m}$ and $v_{odo}^{b}$ can be described as

(14) $$v_{odo}^{m}=C_{b}^{m}v_{odo}^{b}$$

The expression for $C_{b}^{m}$ in the above equation is

(15) $$C_{b}^{m}=I+\left(\alpha \times \right)=\left[\begin{array}{ccc} 1 & -\alpha_{\psi } & \alpha_{\gamma }\\ \alpha_{\psi } & 1 & -\alpha_{\theta }\\ -\alpha_{\gamma } & \alpha_{\theta } & 1 \end{array}\right]$$

Most existing studies treat the odometer scale factor $\delta_{K}$ as a constant. However, as reported by Zou et al. [1], seabed sediments are inherently deformable, and their shear strength varies with depth. Consequently, $\delta_{K}$ cannot be assumed constant, but instead depends on terrain and substrate properties, such as internal friction angle and cohesion. Ignoring this variability may introduce significant nonlinear errors, particularly when transitioning between different substrates (e.g., from hard ground to soft mud).

To characterize the stochastic variations induced by terrain deformation and complex robot–ground interactions, the scale factor error is modeled as a first-order Gauss–Markov process:

(16) $$\dot{\delta x}(t)=-\frac{1}{\tau }\delta x(t)+\omega \left(t\right)$$

where δx(t) represents the time-varying error (here, the odometer scale factor deviation $\delta_{K}$), τ is the time constant, which characterizes how quickly the error tends to decay toward zero, and $\omega \left(t\right)$ is a zero-mean white Gaussian noise term that introduces stochastic perturbations.

#### 2.2.4. Construction of High-Dimensional Error State Space Model

Optimal estimation of integrated navigation system errors within the Kalman filter framework requires first establishing a linearized state equation describing error evolution. To account for seabed-induced sensor inaccuracies and the time-varying odometry scale factor, the odometer calibration error is incorporated into the conventional 15-dimensional SINS error state, resulting in a 16-dimensional error state vector *X*; to enable recursive estimation, a 16-dimensional error state vector is constructed as

(17) $$X(t)={\left[\begin{array}{cccccc} \varphi^{T} & \delta v^{T} & \delta p^{T} & \varepsilon^{b^{T}} & \sigma^{b^{T}} & \delta K \end{array}\right]}^{T}$$

where $\varphi \in R^{𝟛}$ is the attitude misalignment angle; $\delta v^{n},\delta p^{n}\in R^{𝟛}$ are the velocity error and position error, respectively; $\varepsilon^{b},\sigma^{b}\in R^{𝟛}$ represent the zero bias of the gyroscope and accelerometer, respectively, modeled as a first-order Gaussian Markov process to describe its random drift characteristics; $\delta K\in R^{𝟙}$ is the odometry scaling factor error. Given the complexity of the mechanical properties of seabed sediments, it is regarded as a random walk process for real-time estimation.

Based on the error dynamics equation derived in Section 2.2, the continuous-time system state equation can be expressed as

(18) $$\dot{X}(t)=F(t)X(t)+G(t)W(t)$$

where $F\left(t\right)$ is the system state transition matrix, $G\left(t\right)$ is the noise distribution matrix, and $W\left(t\right)$ is the process noise matrix.

The matrix F(t) exhibits a block structure:

(19) $$\begin{array}{c} F(t)=\left[\begin{array}{cccccc} M_{aa} & M_{av} & M_{ap} & -C_{b}^{n} & 0_{3\times 3} & 0_{3\times 1}\\ M_{va} & M_{vv} & M_{vp} & 0_{3\times 3} & C_{b}^{n} & 0_{3\times 1}\\ 0_{3\times 3} & M_{pv} & M_{pp} & 0_{3\times 3} & 0_{3\times 3} & 0_{3\times 1}\\ 0_{6\times 9} & & {diag(-\frac{1}{\tau_{i}})}_{6\times 6} & & 0_{6\times 1} & \\ 0_{1\times 15} & & & & -\frac{1}{\tau_{k}} & \end{array}\right] \end{array}$$

Here, $M_{xy}$ denotes the corresponding error coupling submatrix, which characterizes the interaction between different state error components in the system; according to the derivation formula of the inertial navigation error equations presented above, the associated relationships can be further obtained as follows:

(20) $$M_{aa}=-(\omega_{in}^{n}\times )$$

(21) $$M_{av}=\left[\begin{array}{ccc} 0 & -\frac{1}{R_{m}+h} & 0\\ \frac{1}{R_{n}+h} & 0 & 0\\ \frac{\tan L}{R_{n}+h} & 0 & 0 \end{array}\right]$$

(22) $$M_{ap}=\left[\begin{array}{ccc} 0 & 0 & \frac{v^{n}}{{\left(R_{m}+h\right)}^{2}}\\ -\omega_{ie}sin\;L & 0 & -\frac{v_{e}^{n}}{{\left(R_{n}+h\right)}^{2}}\\ \omega_{ie}cos\;L+\frac{V_{e}^{n}{sec}^{2}\;L}{R_{n}+h} & 0 & -\frac{v_{e}^{n}tan\;L}{{\left(R_{n}+h\right)}^{2}} \end{array}\right]$$

(23) $$M_{vv}=\left(v^{n}\times \right)M_{1}-\left(\left(2\omega_{ie}^{n}+\omega_{en}^{n}\right)\times \right)$$

(24) $$M_{vp}=\left(v^{n}\times \right)\left[\begin{array}{ccc} 0 & 0 & \frac{v^{n}}{{\left(R_{m}+h\right)}^{2}}\\ -2\omega_{ie}sin\;L & 0 & -\frac{v_{e}^{n}}{{\left(R_{n}+h\right)}^{2}}\\ 2\omega_{ie}cos\;L+\frac{v_{e}^{n}{sec}^{2}\;L}{R_{n}+h} & 0 & -\frac{v_{e}^{n}tan\;L}{{\left(R_{n}+h\right)}^{2}} \end{array}\right]$$

(25) $$M_{pv}=\left[\begin{array}{ccc} 0 & \frac{1}{R_{m}+h} & 0\\ \frac{\sec L}{R_{n}+h} & 0 & 0\\ 0 & 0 & 0 \end{array}\right]$$

(26) $$M_{pp}=\left[\begin{array}{ccc} 0 & 0 & -\frac{v^{n}}{{\left(R_{m}+h\right)}^{2}}\\ \frac{v_{e}^{n}sec\;Ltan\;L}{R_{n}+h} & 0 & -\frac{v_{e}^{n}sec\;L}{{\left(R_{n}+h\right)}^{2}}\\ 0 & 0 & 0 \end{array}\right]$$

(27) $${diag(-\frac{1}{\tau_{i}})}_{6\times 6}=\left[\begin{array}{cc} {-\frac{1}{\tau_{g}}\;I}_{3\times 3} & 0_{3\times 3}\\ 0_{3\times 3} & {-\frac{1}{\tau_{a}}\;I}_{3\times 3} \end{array}\right]$$

$\tau_{i}$ is the relevant time constant of the inertial device. $\tau_{g}$ represents the time constant of the gyroscope, while $\tau_{a}$ represents the time constant of the accelerometer. I represents the identity matrix. $W\left(t\right)$ represents the system noise vector, encompassing the white noise of gyroscope and accelerometer measurements.

### 2.3. Measurement Equations Based on Non-Holonomic Constraint Analysis and Zero-Velocity Update

Building upon the single-system error propagation model, this section addresses the unified state-space representation for multi-source information fusion. Given the “long-duration passive” and “intermittent stop” operational characteristics of seabed drilling robots in deep-sea environments, odometer aiding alone is insufficient to suppress inertial drift. To this end, non-holonomic constraints (NHCs) and zero-velocity updates (ZUPTs) are incorporated to formulate a high-dimensional state-space model encompassing both sensor intrinsic errors and motion-state errors. Measurement equations derived from kinematic constraints are further constructed to effectively mitigate divergence.

#### 2.3.1. Analysis of Non-Integrity Constraints and Zero-Velocity Correction Principle

For an ideal underwater drilling robot, its motion is constrained by the contact with the strata and the structural configuration. In the body coordinate system only the forward direction is allowed to generate effective motion speed, while the lateral and vertical velocity components should theoretically be zero, that is, satisfying the non-integrity motion constraint:

(28) $$v^{b}={\left[\begin{array}{ccc} v_{x}^{b} & v_{y}^{b} & v_{z}^{b} \end{array}\right]}^{T}$$

where $v_{x}^{b}=0$, $v_{z}^{b}=0$. This reflects the fact that the drilling rig cannot produce free-slip velocities laterally or vertically. This constraint is inherently a linear constraint on the velocity space rather than a geometric pose constraint, and is therefore classified as a non-holonomic constraint (NHC). In practice, due to accelerometer bias and attitude errors, lateral and vertical velocity errors accumulate unboundedly in an inertial navigation system. By representing the NHC as a pseudo-observation within the filter, lateral and vertical velocity drift can be effectively suppressed during active motion.

When the robot is stationary—during stop drilling, attitude adjustment, or waiting—the actual velocity in the navigation frame approaches zero:

(29) $$v^{n}=0$$

Zero-velocity updates exploit this high-confidence stationary state to strongly constrain inertial velocity errors, simultaneously correcting accelerometer bias and attitude errors. In essence, NHC primarily constrains the system during continuous motion, while ZUPT applies during stationary or quasi-stationary phases. In complex seabed conditions, integrating NHC, ZUPT, and odometry measurements enables a multi-source fusion framework, providing robust and high-precision navigation under both motion and stationary phases.

#### 2.3.2. Zero-Velocity Update Measurement Model

During drilling, sampling, or communication standby, the robot chassis remains effectively stationary relative to the seabed. Under these conditions, the true velocity measured by the navigation system should theoretically be zero. As noted in [17], this physical fact can be exploited to construct “zero-velocity observations,” effectively halting the integral accumulation of velocity errors and enhancing the observability of IMU bias. The corresponding ZUPT measurement equation is then formulated as follows:

(30) $$Z_{ZUPT}=v_{SINS}^{n}-0=H_{ZUPT}X+V_{ZUPT}$$

where the measurement matrix $H_{ZUPT}$ directly selects the velocity error term in the state vector:

(31) $$H_{ZUPT}=\left[\begin{array}{ccc} 0_{3\times 3} & I_{3\times 3} & 0_{3\times 10} \end{array}\right]$$

Although the ZUPT principle is straightforward, complex sea conditions—such as ocean currents—can induce small chassis vibrations. Consequently, robust techniques, such as the chi-square test, are required to dynamically adjust the ZUPT trigger threshold, preventing micro-movements from being misclassified as a stationary state.

#### 2.3.3. Non-Integrity Constraint (NHC) Measurement Model

During maneuvering drilling, the robot is assumed to exhibit negligible lateral slip and vertical displacement, approximately satisfying non-holonomic constraints. In the body-fixed coordinate system, the lateral velocity $v_{x}^{b}$ and vertical velocity $v_{z}^{b}$ can thus be considered near zero.

Based on this assumption, the NHC measurement equation is constructed. First, the velocity is calculated using SINS. $v_{SINS}^{n}$ is projected onto the carrier coordinate system and compared with the virtual constraint value ${\left[0,v_{y},0\right]}^{T}$, leading to

(32) $$Z_{NHC}=C_{n}^{b}v_{SINS}^{n}-\left[\begin{array}{c} 0\\ v_{odo}^{b}\\ 0 \end{array}\right]\approx \left[\begin{array}{c} \delta v_{x}^{b}\\ \delta v_{y}^{b}\\ \delta v_{z}^{b} \end{array}\right]$$

Considering the influence of lever arm effect and installation error angle, the linearized NHC measurement matrix is derived, $H_{NHC}$. In the derivation process, the coupling term of attitude error to velocity projection needs to be retained to ensure the consistency of filtering. After expansion, we get

(33) $$Z_{NHC}=H_{NHC}X+V_{NHC}$$

(34) $$H_{NHC}=\left[\begin{array}{ccccc} \left(C_{n}^{b}v^{n}\times \right) & C_{n}^{b} & 0_{3\times 3} & 0_{3\times 6} & J_{\delta K} \end{array}\right]$$

where $\left(C_{n}^{b}v^{n}\times \right)$ is the antisymmetric matrix formed by the velocity vectors, which reflects the influence of attitude error on velocity observation; $J_{\delta K}$ is the Jacobian term of odometer scale error.

Since the output of strapdown inertial navigation contains the velocity and position information of the carrier, the obtained velocity and position calculation results and the SINS output value can be used to construct the observation vector of the integrated navigation system. The measurement equation is

(35) z=Hx+V

where $H=\left[\begin{array}{ccc} H_{ZUPT} & H_{NHC1} & H_{NHC2} \end{array}\right]$.

Define vectors $E_{1}$, $E_{2}$ and $E_{3}$:

(36) $$E_{1}=\left[\begin{array}{c} 1\\ 0\\ 0 \end{array}\right],E_{2}=\left[\begin{array}{c} 0\\ 1\\ 0 \end{array}\right],E_{3}=\left[\begin{array}{c} 0\\ 0\\ 1 \end{array}\right]$$

as the observations constructed with zero-velocity correction. The observation matrix obtained for zero-velocity correction is $H_{ZUPT}$ as

(37) $$H_{ZUPT}=\left[0_{3\times 3}\;I_{3\times 3}\;0_{3\times 12}\right]$$

Regarding the solution of $H_{NHC1}$ and $H_{NHC2}$, define $A_{x,zGR}$ as the carrier acceleration on the x–z plane derived from the gyroscope measurements $\omega_{eb}^{n}$. The *y*-axis acceleration of the carrier in the navigation frame, $A_{yAC}^{n}$, is obtained from the triaxial accelerometer. Additionally, $A_{y-odo}$ denotes the y-axis acceleration measured by the odometer. These tangential and normal acceleration components from both the accelerometer and odometer are employed as observations for constructing the NHC measurement equations.

The first observation is obtained by subtracting the tangential acceleration:

(38) $$Z_{1}={\tilde{A}}_{yAC}^{n}-{\tilde{A}}_{yodo}^{n}={\tilde{C}}_{b}^{n}\left[\begin{array}{ccc} 0 & E_{2} & 0 \end{array}\right]{\tilde{C}}_{n}^{b}{\tilde{F}}_{en}^{n}-{\tilde{C}}_{b}^{n}E_{2}\frac{d{\tilde{V}}_{odo}}{dt}|b$$

where $F_{en}^{n}=\frac{dV_{en}}{dt}{|}_{b}^{n}+\omega_{eb}^{n}\times V_{en}^{n}$, ${\tilde{A}}_{yAC}^{n},{\tilde{A}}_{yOD}^{n},{\tilde{C}}_{b}^{n},{\tilde{C}}_{n}^{b},{\tilde{F}}_{en}^{n},{\tilde{v}}_{odo}$ is the corresponding quantity with error value in the actual experiment.

(39) $${\tilde{C}}_{b}^{n}=\left[\begin{array}{c} I-\left(\varphi \times \right) \end{array}\right]C_{b}^{n}$$

(40) $${\tilde{C}}_{n}^{b}=C_{n}^{b}\left[I+\left(\varphi \times \right)\right]$$

(41) $${\tilde{F}}_{en}^{n}=\left(\begin{array}{c} F_{en}^{n}+{\tilde{C}}_{b}^{n}\sigma^{b} \end{array}\right)$$

(42) $${\tilde{v}}_{odo}=\left(\begin{array}{c} 1+\delta k \end{array}\right)v_{odo}$$

We can obtain the corresponding expressions:

(43) $$H_{NHC1}=\left[H_{11}\;0_{3\times 3}\;0_{3\times 3}\;aC_{b}^{n}\;0_{3\times 3}-C_{b}^{n}E_{2}\frac{dv_{odo}}{dt}{|}_{b}\right]$$

where $a=C_{b}^{n}\left[0\;E_{2\;}\;0\right]C_{n}^{b}$, $H_{11}=-a\left(F_{en}^{n}\times \right)$.

In the same way, the observation of the normal direction can be obtained:

(44) $$Z_{2}={\tilde{A}}_{x,zAC}^{n}-{\tilde{A}}_{x,zGR}^{n}={\tilde{C}}_{b}^{n}\left[\begin{array}{ccc} E_{1} & 0 & E_{3} \end{array}\right]{\tilde{C}}_{n}^{b}{\tilde{F}}_{en}^{n}-{\tilde{\omega }}_{eb}^{n}\times {\tilde{V}}_{en}^{n}$$

We can obtain:

(45) $$H_{NHC2}=\left[\begin{array}{cccccc} H_{21} & -\left(\omega_{eb}^{n}\times \right) & 0_{3\times 3} & bC_{b}^{n} & \left(V_{en}^{n}\times \right)C_{b}^{n} & 0 \end{array}\right]$$

where $b=C_{b}^{n}\left[E_{2}\;0\;E_{3}\right]C_{n}^{b}$.

Notably, NHCs can fail under rugged seabed terrain or strong lateral currents. As shown in [18], forcibly constraining lateral velocity to zero introduces model bias. To address this, the proposed algorithm avoids rigid enforcement and instead adaptively adjusts the measurement noise covariance matrix by monitoring the residual statistics $R_{NHC}$. When significant slippage or lateral displacement occurs, the measurement noise variance in the affected dimension is increased, enabling a smooth transition from “hard” to “soft” constraints.

## 3. Design of High-Precision Robust Navigation Algorithm

Operating in the high-pressure deep-sea environment, the seabed drilling robot is subjected to non-Gaussian noise and sensor observation anomalies. Traditional extended Kalman filters (EKFs) often suffer divergence due to linearization truncation errors, while cubature Kalman filters (CKFs) may fail to maintain covariance matrix positive definiteness over long-term operation [25,26,27,28]. To address these challenges, this work proposes a navigation framework based on the robust square-root cubature Kalman filter. By preserving the square-root form of the covariance matrix via *QR* decomposition and integrating an anomaly detection mechanism using composite cable encoder data, the algorithm achieves enhanced numerical stability and adaptive suppression of non-line-of-sight errors and slippage disturbances.

### 3.1. Robust Square-Root Volume Kalman Filter Theory

Time Update:

Assume k the posterior state estimate at time $\hat{x_{k|k}}$ is $S_{k|k}$. Based on the third-order sphere diameter-volume criterion, generate 2n cubature points:

(46) $$x_{i,k|k}=S_{k|k}\xi_{i}+{\hat{x}}_{k|k},i=1,2,\ldots ,2n$$

where $\xi_{i}$ is the basic volume point set, defined as $\xi_{i}=\sqrt{n}{\left[I\right]}_{i}\;(i=1\;\ldots n)$ and $\xi_{i}=-\sqrt{n}{\left[I\right]}_{i-n}(i=n+1\;\ldots 2n$).

Substitute the volume points into the nonlinear state transition equation $f\left(\cdot \right)$ for propagation:

(47) $$x_{i,k+1|k}^{*}=f\left(x_{i,k|k}\right)$$

Calculate the one-step state prediction value ${\hat{x}}_{k+1|k}$ and the square-root factor of the prediction covariance $S_{k+1|k}$. Numerical stability is guaranteed by *QR* decomposition and triangulation update operators:

(48) $${\hat{x}}_{k+1|k}=\frac{1}{2n}\sum_{i=1}^{2n}\;x_{i,k+1|k}^{*}$$

The characteristic square root of the prediction error covariance matrix is

(49) $$S_{k+1|k}=Tria\left(\left[x_{1:2n,k+1|k}^{*}-{\hat{x}}_{k+1|k},S_{Q,k}\right]\right)$$

where $S_{Q,k\;}$ is the square-root factor of the process noise covariance matrix $Q_{k}$.

After passing through the measurement function h, the set of cubature points after propagation of the nonlinear measurement function is

(50) $$z_{i,k+1|k}=h\left(x_{i,k+1|k}\right)$$

At this time, the square root of the measurement prediction value, the innovation, and the innovation covariance matrix are

(51) $${\hat{z}}_{k+1|k}=\omega_{i}\sum_{i=1}^{2n}\;z_{i,k+1|k}$$

(52) $$\left\{\begin{array}{l} \eta_{k+1}=z_{k+1}-{\hat{z}}_{k+1|k}\\ S_{ZZ,k+1|k}=Tria(\left[\begin{array}{c} D_{Z_{k+1|k}},S_{R} \end{array}\right]) \end{array}\right.$$

The cross-covariance matrix is

(53) $$P_{XZ,k+1|k}=D_{X_{k+1|k}}\times D_{Z_{k+1|k}}^{T}$$

At time *k* + 1, the characteristic square roots of the error variance of the filter gain state estimate are

(54) $$K_{k+1}=\frac{P_{XZ,k+1|k}}{S_{ZZ,k+1|k}S_{ZZ,k+1|k}^{T}}$$

(55) $$S_{k+1|k+1}=Tria\left(\left[D_{X_{k+1|k}}-K_{k+1}D_{Z_{k+1|k}},K_{k+1}S_{R}\right]\right)$$

### 3.2. Odometry-Based Anomaly Detection and Adaptive Compensation Mechanism

The seabed drilling robot operates in a distinctive mode: it initially penetrates the strata with seabed base station assistance, then drills autonomously using its own drive mechanism. During descent, the tail composite cable—responsible for power, communication, and traction—unwinds from the base station. An encoder mounted on the winch or cable port provides relative position observations by recording the cable release length.

In practice, encoder measurements are highly susceptible to environmental and operational conditions, primarily manifesting as follows:

1. Cable slack or tension effect: Ocean currents can induce nonlinear cable deformations, causing encoder-measured cable length to deviate from the robot’s true displacement.
2. Drilling slippage: When the drill encounters hard rock or torque is insufficient, the robot may idle or move minimally. If the cable is passively pulled by ocean currents, false odometer increments result.

These anomalies violate the Gaussian white noise assumption for observation innovations, potentially contaminating state estimation if uncorrected. To address this, a chi-square-based fault detection mechanism with adaptive variance inflation is implemented to ensure robust filtering.

Anomaly detection statistics construction: Using the measurement innovation $\eta_{k+1}=z_{k+1}-{\hat{z}}_{k+1|k}$ and its theoretical covariance $P_{zz,k+1}=S_{zz,k+1}S_{zz,k+1}^{T}$, the squared Mahalanobis distance is constructed as the detection statistic $M_{k}^{2}$:

(56) $$M_{k}^{2}=\eta_{k+1}^{T}(P_{zz,k+1}{)}^{-1}\eta_{k+1}$$

Under normal operating conditions, $M_{k}^{2}$ follows a chi-square distribution with m degrees of freedom ($\chi_{m}^{2}$). Set the significance level as α (e.g., 0.05) and the corresponding decision threshold as $\chi_{m,\alpha }^{2}$.

To mitigate the influence of outliers, an adaptive residual weighting mechanism based on the Mahalanobis distance is introduced. When $M_{k}^{2}>\chi_{m,\alpha }^{2}$, it is determined that the current composite cable encoder data is abnormal (e.g., slippage or external disturbance). At this time, a robust adaptive factor $\lambda_{k}$ is introduced to expand the measurement noise covariance $R_{k}$ in real time to reduce the filtering gain of abnormal measurements at this moment.

Adaptive factors are constructed using a two-segment IGG weight function:

(57) $$\lambda_{k}=\left\{\begin{array}{l} 1,M_{k}^{2}\leq \chi_{m,\alpha }^{2}\;(normal)\\ \frac{M_{k}^{2}}{\chi_{m,\alpha }^{2}},\;M_{k}^{2}>\chi_{m,\alpha }^{2}\;(abnormal,\;weighted\;down) \end{array}\right.$$

The corrected square root of the measurement noise covariance ${\tilde{S}}_{R,k}$ is updated to

(58) $${\tilde{S}}_{R,k}=\sqrt{\lambda_{k}}\cdot S_{R,k}$$

By increasing R the filter automatically “discounts” the current encoder measurements, relying more heavily on inertial system predictions. This strategy preserves trajectory smoothness and robustness during cable slippage or other disturbances.

In summary, the pseudocode of the proposed robust square-root cubature Kalman filter is presented below. The algorithm structure delineates the full logic from inertial prediction to robust correction, ensuring reliable navigation under complex seabed drilling conditions.

The RSRCKF algorithm is illustrated by the pseudocode in Algorithm 1.
**Algorithm 1.** Square-root cubature Kalman filter**Input***:* $S_{0}$, $S_{Q}$, $S_{R}$, $T_{th}$**Output***:* ${\hat{x}}_{k|k}$, $S_{k|k}$1*:*        *Setting number of cycles N*2*:*        *Filter initialization*3*:*        $S_{0|0}=S_{0},\;{\hat{x}}_{0|0}={\hat{x}}_{0}$
4*:*        **while**
*k* ≤ *N* **do**5*:*        Time update6*:*           $x_{i,k-1|k-1}=S_{k-1|k-1}\xi_{i}+{\hat{x}}_{k-1|k-1}$, $x_{i,k|k-1}^{*}=f\left(x_{i,k-1|k-1}\right)$
7*:*           ${\hat{x}}_{k|k-1}=\frac{1}{2n}\sum_{i=1}^{2n}\;x_{i,k|k-1}^{*}$
8*:*           $S_{k|k-1}=Tria\left(\left[x_{1:2n,k|k-1}^{*}-{\hat{x}}_{k|k-1},S_{Q,k-1}\right]\right)$
9*:*        Measurement update10*:*         $z_{i,k|k-1}=h(x_{i,k|k-1})$
11*:*         ${\hat{z}}_{k|k-1}=\frac{1}{2n_{x}}\sum Z_{i,k|k-1}$
12*:*         $\eta_{k}=z_{k}-{\hat{z}}_{k|k-1}$
13*:*         $S_{zz}=qr\{[z_{k|k-1}-{\hat{z}}_{k|k-1},S_{R}{]}^{T}\}$
14*:*         $M_{k}^{2}=\eta_{k}^{T}(S_{zz}S_{zz}^{T}{)}^{-1}\eta_{k}$
15*:*      **Switch**16*:*         **if** $M_{k}^{2}>T_{th}$ **then** $\lambda_{k}=M_{k}^{2}/T_{th}$
17*:*         ${\bar{S}}_{R}=\sqrt{\lambda_{k}}S_{R}$
18*:*         $S_{zz}=qr\{[z_{k|k-1}-{\hat{z}}_{k|k-1},{\bar{S}}_{R}{]}^{T}\}$
19*:*       **else** ${\bar{S}}_{R}=S_{R}$
20*:*       **end if**21*:*         $K_{k}=P_{xz}(S_{zz}S_{zz}^{T}{)}^{-1}$
22*:*         ${\hat{x}}_{k|k}={\hat{x}}_{k|k-1}+K_{k}\eta_{k}$
23*:*         *k* ← *k* + 124*:*       **end while**

The algorithm architecture delineates the full workflow from inertial prediction to robust correction, ensuring reliable system performance under complex seabed drilling conditions.

## 4. Simulation and Experimental Analysis

To evaluate the navigation performance of the proposed robust square-root cubature Kalman filter in complex seabed environments, a two-stage validation approach is adopted: numerical simulation and physical experimentation. The simulation stage assesses algorithm robustness under slippage and non-Gaussian noise, while the experimental stage employs the independently developed seabed drilling robot to conduct field tests in tidal flats near a seaside wharf, thereby demonstrating practical applicability in real-world conditions.

### 4.1. Numerical Simulation Analysis

To replicate typical seabed drilling conditions on a soft mud substrate, a high-fidelity simulation environment is developed in MATLAB (v2024). Following relevant studies in deep-sea navigation, the sensor parameters for the strapdown inertial navigation system (SINS) and odometry (OD) are configured as listed in Table 1. The system’s initial position is set to (30° N, 122° E), with initial attitude errors of [0.1°, 0.1°, 1°].

A 1500 s figure-eight trajectory, incorporating straight segments, was designed to simulate the robot’s roving exploration and drilling operations on the seabed. To evaluate the algorithm’s capability in addressing slippage, an abrupt odometry scaling factor change was introduced between 400 and 1200 s of the simulation, representing severe slippage in soft mud (scaling factor error jumping from 2% to 20%), along with non-Gaussian measurement noise.

Four algorithms were selected for comparative analysis:

EKF: Traditional EKF serving as a benchmark, offering only basic linearization.

SRCKF: Standard SRCKF with strong nonlinear handling, but lacking robustness mechanisms.

HKF (Huber base KF): A robust Kalman filter incorporating the Huber M-estimation to suppress the influence of outliers, but still limited by the linearization framework of the EKF.

Proposed RSRCKF: Robust square-root cubature Kalman filter incorporating chi-square-based fault detection and adaptive variance inflation, as proposed in this work.

**Figure 3:**
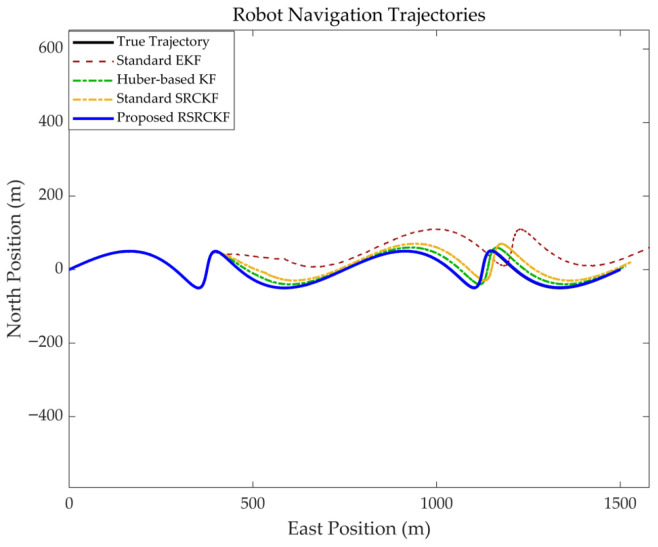
Comparison of trajectory and theoretical trajectory under three algorithms.

As illustrated by the planar trajectory comparison in Figure 3, all four algorithms accurately track the reference trajectory during nominal operation (0–400 s). Upon entering the slippage interval at 400 s, where the odometer-derived velocity significantly exceeds the true motion, the EKF (pink) rapidly diverges and deviates from the intended path. The standard SRCKF (yellow), although partially mitigating nonlinear effects via the cubature rule, remains vulnerable to corrupted observations due to the absence of an anomaly detection mechanism, resulting in pronounced trajectory drift. The HKF (green), by incorporating Huber M-estimation, reduces the influence of outliers through adaptive residual weighting and thus exhibits improved robustness compared to the SRCKF. However, its performance still degrades under sustained slippage conditions, leading to noticeable trajectory deviations.

In contrast, the proposed RSRCKF (blue) monitors the residual Mahalanobis distance $M_{k}^{2}$ in real time, enabling reliable detection of abnormal measurements during slippage. By adaptively inflating the measurement noise covariance $R_{k}$ the filter effectively down-weights odometer contributions and maintains close agreement with the ground truth.

**Figure 4:**
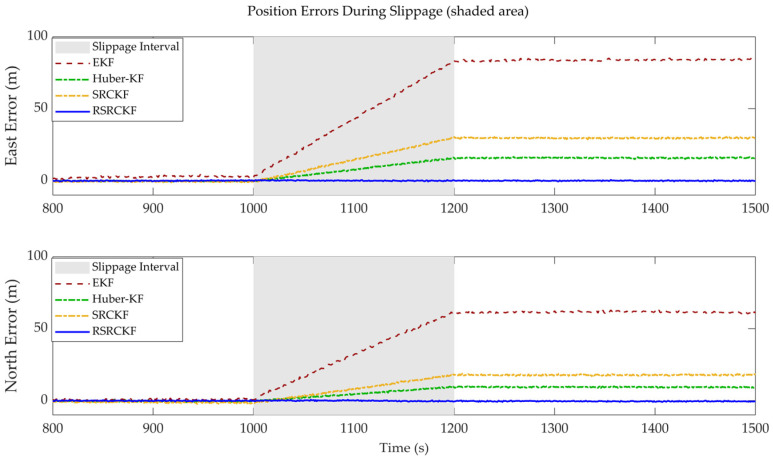
Changes in position error over time in the east and north directions.

Figure 4 further illustrates the changes in position error over time in the east and north directions. The EKF exhibits clear divergence after slippage onset, with errors exceeding 50 m. The SRCKF accumulates substantial errors within the slippage interval, and the resulting deviation persists even after normal conditions resume. The HKF shows improved robustness compared to the SRCKF by suppressing the influence of outliers through Huber-based residual weighting. However, in the presence of sustained slippage, its performance still degrades, with errors remaining at approximately 10 m, indicating limited capability in handling prolonged abnormal measurements.

In contrast, the RSRCKF maintains minimal error fluctuations throughout the slippage interval (gray shaded region), with a maximum error below 1.5 m. This performance is attributed to the adaptive robustness factor $\lambda_{k}$, which enables the filter to effectively discount unreliable odometer measurements and rely more heavily on short-term, high-accuracy SINS predictions, thereby demonstrating strong robustness in soft mud environments.

**Figure 5:**
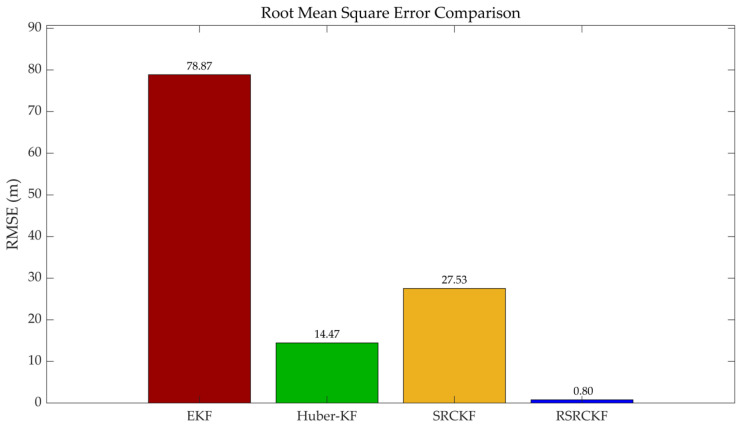
Comparison of overall root mean square error of four algorithms.

To quantitatively evaluate the accuracy of each algorithm, the root mean square error (RMSE) over the entire trajectory is analyzed, as shown in Figure 5. The results indicate that the conventional EKF exhibits the largest error (78 m), while the HKF and SRCKF reduce the RMSE to 14 m and 28 m, respectively, demonstrating partial improvement under non-ideal conditions. In contrast, the proposed RSRCKF achieves a significantly lower RMSE of 0.80 m, indicating a substantial enhancement in positioning accuracy compared to the other methods.

### 4.2. Actual Test on Beach Pier and Mudflat

To validate the engineering applicability of the proposed algorithm, field experiments were conducted in the tidal flat area of Zhoushan Wharf, supported by the “Natural Gas Hydrate Pilot Production Area Formation Spatial Drilling Monitoring System” project. The test site is predominantly composed of saturated soft clay and silt, whose mechanical properties closely resemble those of shallow subsea sediments, thereby providing a realistic proxy for the operational environment and disturbance conditions encountered by seabed drilling robots.

**Figure 6:**
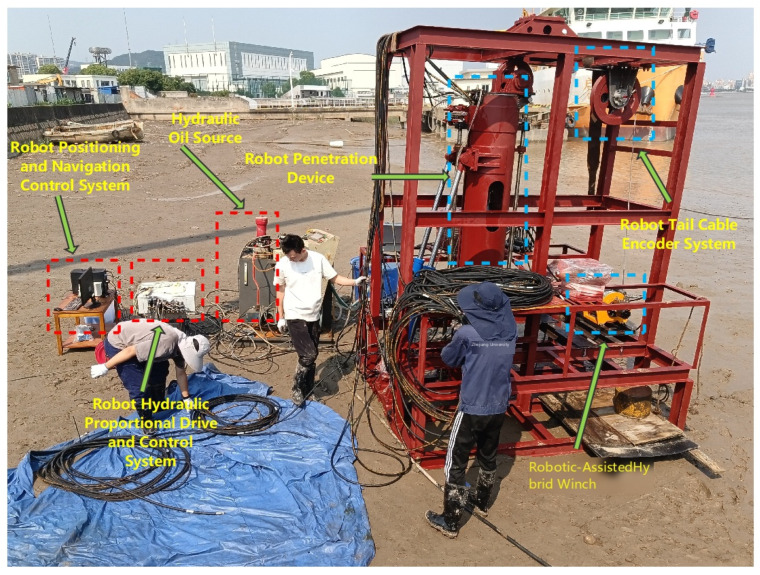
Robot and base station system test on the wharf tidal flat.

The experimental platform comprises a self-developed modular seabed drilling robot and a seabed base station (as shown in Figure 6). The robot is approximately 2.5 m in length and 0.22 m in diameter, with a mass of about 150 kg, while the base station measures 2.5 m × 2.5 m × 3 m.

The navigation sensor suite consists of a self-developed high-precision MEMS inertial measurement unit (IMU), an odometer (OD), and a high-precision depth sensor. Among these, the depth sensor serves only as an auxiliary sensor and does not participate in the navigation data processing. The navigation experimental system primarily comprises the self-developed high-precision MEMS strapdown inertial navigation system (SINS) and the odometer. Specifically, the MEMS SINS is mounted on the drilling robot and outputs data at 100 Hz. Its MEMS gyroscopes have a full scale of ±300°/s with a bias stability better than 5°/h, and its MEMS accelerometers have a full scale of ±50 g with a bias stability better than 0.5 mg. The odometer is installed on the seafloor base station to monitor the real-time deployment and retrieval length of the rear composite cable, providing equivalent odometry information at 10 Hz. The high-precision depth sensor, mounted on the base station, is used solely to measure water depth. During operation, IMU data, odometer measurements, and depth sensor readings are synchronously recorded, while offline navigation algorithm validation is performed based on the IMU and odometer data. The initial position for the experiment is obtained from the base station’s own positioning system.

The experimental procedure for the drilling robot is as follows:

MEMS Strapdown Inertial Navigation System (SINS): Perform gyroscope and accelerometer bias calibration, alignment, and other initialization procedures.

Encoder Odometer: Set the zero-reference point and verify the correctness of the length counting during extension and retraction.

Depth Sensor: Perform zero-point calibration to ensure the accuracy of auxiliary data.

1. Pre-experiment check: Inspect both hardware and software for any anomalies. Once confirmed normal, install the drilling robot, seafloor base station, and all related equipment in their designated positions.
2. Connection and power-on: Connect all system wiring and the power supply, and power on the inertial navigation system, odometer encoder, and depth sensor.
3. Sensor initialization and parameter adjustment: After powering on, initialize the sensors and adjust relevant parameters:
4. Data acquisition and experiment start: Once initialization and parameter adjustment are completed, simultaneously collect data from the SINS, odometer encoder, and depth sensor, and prepare to begin the experiment.
5. During the experiment: Continuously monitor the sensor data for anomalies and record observations.

The experiment was conducted on a muddy tidal flat under low-tide conditions, performing approximately 600 s of directional drilling. The starting position of the robot was set as the origin, and it drilled diagonally downward along a straight path at a 45° angle, with the preset trajectory serving as the theoretical reference for the true trajectory. To obtain the actual operational trajectory, experimenters manually measured the robot’s position by inserting a long ruler into the sediment. During manual measurement, the readings corresponded to the robot’s outer shell, which has a fixed offset from the inertial navigation center and therefore required software compensation. For the 45° drilling, both vertical and horizontal compensation values were 3.54 cm, effectively eliminating systematic errors. Measurement errors primarily depended on the ruler’s precision: using a high-precision ruler with an accuracy of ±0.5 mm and taking the median of five repeated measurements, the deviation from the true position could be controlled within ±1 mm, making it sufficient as a reference for the true trajectory. During the experiment, both inertial navigation and odometer data were collected and subsequently processed using three methods: pure SINS inertial navigation, standard SINS/OD integrated navigation, and the RSRCKF integrated navigation algorithm proposed in this study.

**Figure 7:**
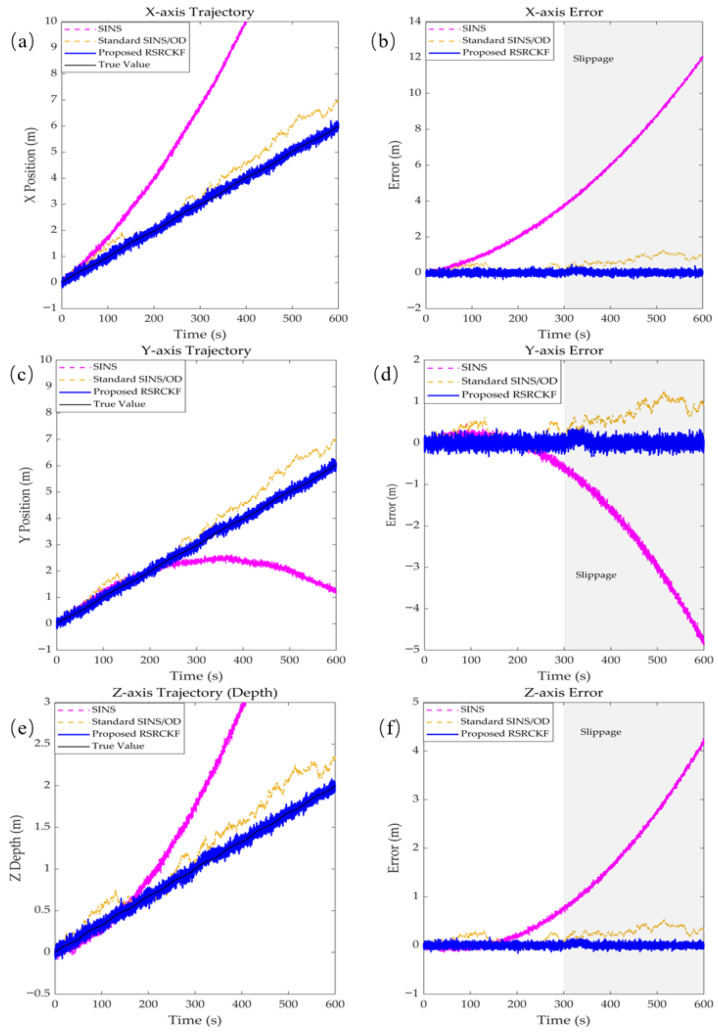
Comparison of three navigation algorithms with actual ground truth trajectories and errors: (**a**) *x*-axis trajectory; (**b**) *x*-axis error; (**c**) *y*-axis trajectory; (**d**) *y*-axis error; (**e**) *z*-axis trajectory; (**f**) *z*-axis error.

Figure 7 compares the estimated trajectories with the ground truth reference. Owing to intrinsic IMU error accumulation, the pure SINS solution exhibits pronounced drift after approximately 200 s. Although the conventional SINS/OD integration mitigates divergence to some extent, it fails to reject spurious displacement induced by slippage; consequently, a significant trajectory deviation arises as the robot traverses the soft mud region (around *T* ≈ 300 s). In contrast, the proposed RSRCKF maintains the highest consistency with the reference trajectory throughout the experiment. To quantitatively evaluate the performance of the proposed method, the root mean square error (RMSE) is adopted as the primary metric. The RMSE is computed based on the position errors with respect to the reference trajectory. The RMSE values reported in Table 2 were calculated over the entire trajectory. Quantitative evaluation within the 10 m × 10 m core test area shows that the positioning error of the proposed method was reduced by about 83% (average).

## 5. Discussion

The aim of this study is to address navigation divergence caused by slippage and sensor anomalies in seafloor drilling robots operating on soft seabeds. Numerical simulations and field experiments conducted on the Zhoushan mudflats validate the effectiveness of the proposed robust square-root cubature Kalman filter (RSRCKF) under complex operating conditions. This section further discusses the core advantages of the algorithm, as well as its potential limitations and directions for future improvement.

1. The experimental results (Figure 3 and Figure 4) indicate that when the robot enters a soft mud region and experiences severe slippage, the positioning errors of both the conventional extended Kalman filter (EKF) and the standard square-root cubature Kalman filter increase approximately linearly, or even diverge over time. This behavior arises because the odometer produces erroneous large displacement measurements under slippage, which are incorrectly interpreted by standard filters as true motion. As a result, the SINS solution is improperly corrected, causing the estimated trajectory to deviate significantly from the ground truth. In contrast, the proposed RSRCKF algorithm continuously monitors the Mahalanobis distance of the innovation vector $\left(M_{k}^{2}\right)$, thereby capturing discrepancies between the observations and model predictions. When slippage occurs, $\left(M_{k}^{2}\right)$ exceeds the chi-square threshold, ($\chi_{m,\alpha }^{2}$), which triggers the robust adaptation mechanism. The algorithm then employs a two-stage IGG weighting function to inflate the measurement noise covariance matrix ($R_{k}$), effectively down-weighting the reliability of the current odometer measurements. This adaptive switching from “hard fusion” to “soft isolation” is the core reason why the algorithm can maintain meter-level positioning accuracy even under strong slip conditions.
2. In the field experiment lasting up to 600 s (Figure 7), the proposed RSRCKF outperforms the standard SINS/OD integrated navigation in terms of positioning accuracy. This performance gain is primarily attributed to the adoption of a square-root filtering architecture. In long-duration operations such as seabed drilling, the accumulation of numerical round-off errors can lead to the loss of positive definiteness of the covariance matrix—particularly in high-dimensional state spaces—ultimately resulting in filter divergence. The RSRCKF propagates the square-root factor of the error covariance matrix ($S_{k|k}$), and performs state recursion using *QR* decomposition and Cholesky updates, thereby inherently preserving the positive semidefiniteness and numerical stability of the covariance matrix. This property is especially critical for state estimation in deep-sea environments characterized by high dynamics and weak observability.
3. Although the proposed method demonstrates excellent performance in a single soft mud substrate, several limitations remain, which point to directions for future research:

Handling prolonged severe slippage: The robustness of the proposed method relies on the assumption that the SINS solution maintains high accuracy over short time intervals. However, if the robot experiences continuous slippage over extended periods (e.g., tens of minutes in fluid-plastic silt), the accumulated drift of the SINS cannot be effectively corrected. Future work may draw on [29,30] to incorporate deep learning-assisted slippage prediction models, establishing a nonlinear mapping between seabed properties and slip rate. This would enable direct compensation of the odometer scale factor, rather than relying solely on measurement down-weighting.

Multi-source heterogeneous fusion: The current system primarily relies on SINS, odometry (OD), and depth measurements. In highly turbid environments, although vision-based methods and Doppler Velocity Logs (DVLs) are often degraded, acoustic positioning systems such as Long Baseline (LBL) or Ultra-Short Baseline (USBL) can provide absolute position updates. The Factor Graph Optimization (FGO) framework proposed in [31,32] offers inherent advantages for handling multi-rate, asynchronous sensor fusion and loop-closure constraints, making it a promising alternative to conventional Kalman filtering architectures.

Refinement of motion constraints: The non-holonomic constraints (NHCs) employed in this study are relatively simplified. Future work could incorporate more comprehensive dynamic constraints by integrating hydrodynamic models and accounting for ocean current disturbances, thereby improving the adaptability of the navigation system under complex marine conditions.

Overall, the proposed RSRCKF provides an effective engineering solution for robust navigation of seabed drilling robots. Nevertheless, further development toward intelligent modeling and full-source sensor fusion is essential to achieve all-weather autonomous operation in deep-sea environments.

## 6. Conclusions

This paper presents a robust and adaptive navigation method for seabed drilling robots operating in soft sedimentary environments. To address the mismatch between odometer measurements and actual motion caused by slippage, a time-varying scale factor is incorporated into the error state model to better capture slippage-induced stochastic errors.

Building upon this model, a robust square-root cubature Kalman filter (RSRCKF) is developed. By leveraging a square-root filtering framework with QR decomposition, the proposed method ensures numerical stability and preserves the positive definiteness of the covariance matrix under nonlinear and non-Gaussian conditions. Furthermore, an adaptive variance inflation mechanism based on a modified chi-square test and a two-stage IGG weighting function are introduced to enable real-time detection and down-weighting of slippage-induced outliers.

Field experiments conducted on the Zhoushan tidal mudflats demonstrate that the proposed method effectively mitigates the impact of false displacement caused by severe slippage. Compared with the conventional SINS/OD integrated navigation scheme, the proposed approach achieves an approximate 82.4% reduction in positioning error, showing significant improvements in both robustness and accuracy.

These results indicate that the proposed RSRCKF-based framework provides a reliable and practical solution for navigation in complex soft seabed environments. Future work will focus on extending the framework to prolonged slippage conditions, incorporating multi-source heterogeneous sensing, and refining motion constraints for improved adaptability in dynamic deep-sea scenarios.

## Figures and Tables

**Table 1:** Simulation sensor parameter settings.

Sensor Type	Parameter Indicators	Parameter Values
Gyroscope	Random walk noise	0.02 (${}^{\circ}/\sqrt{h}$)
	Drift or bias	0.05 (°/h)
Accelerometer	Random walk noise	$10^{-6}(g\cdot \sqrt{s})$
	Drift or bias	20(μg)
Odometer	Scale factor error	20 (ppm)
	Speed measurement noise (standard deviation)	0.05 (m/s)
Simulation environment	Sampling frequency	100 Hz (SINS)
		10 Hz (OD)

**Table 2:** RMSE results.

Method	*X* (m)	*Y* (m)	*Z* (m)
Standard SINS/OD	0.612	0.605	0.314
Proposed RSRCKF	0.102	0.101	0.061

## Data Availability

The original contributions presented in this study are included in the article. Further inquiries can be directed to the corresponding authors.
